# Barriers to delirium screening and management during hospital admission: a qualitative analysis of inpatient nursing perspectives

**DOI:** 10.1186/s12913-023-09681-4

**Published:** 2023-06-29

**Authors:** Jacqueline Ragheb, Alexandra Norcott, Lakeshia Benn, Nirav Shah, Amy McKinney, Lillian Min, Phillip E. Vlisides

**Affiliations:** 1 Department of Anesthesiology, University of Michigan Medical School, 1H247 UH, 1500 East Medical Center Drive, Ann Arbor, MI SPC-5048, 48109-5048 USA; 2 Department of Internal Medicine, Division of Geriatric & Palliative Medicine, Michigan Medicine, Ann Arbor, MI USA; 3 Department of Inpatient Rehabilitation, Michigan Medicine, Ann Arbor, MI USA; 4 College of Health Professions & McAuley School of Nursing, University of Detroit Mercy, Detroit, MI USA; 5 VA Ann Arbor Healthcare System, Department of Internal Medicine, Division of Geriatric Research, Education, and Clinical Centers (GRECC), Ann Arbor, MI USA; 6 Center for Consciousness Science, University of Michigan Medical School, Ann Arbor, MI USA

**Keywords:** Decision support (clinical), Evaluation methodology, Implementation science, Quality Improvement

## Abstract

**Background:**

Delirium in hospitalized patients is a major public health issue, yet delirium is often unrecognized and missed during inpatient admission. The objective of this study was to identify barriers to delirium screening, identification, and management from a nursing perspective on inpatient, acute care units.

**Methods:**

This was a pre-implementation, diagnostic evaluation study to determine current practice patterns and potential barriers to optimizing delirium care at a major university hospital. A qualitative approach was used, which included focus groups of inpatient nurses working on major medical and surgical acute care units. Focus groups were conducted until signs of thematic saturation were present, and data were analyzed via inductive thematic analysis, without predetermined theories or structures. A consensus approach was utilized for transcript coding, and final themes were generated after multiple reviews of initial themes against transcript datasets.

**Results:**

Focus group sessions (n = 3) were held with 18 nurses across two major inpatient units. Nurses reported several barriers to successful delirium screening and management. Specific challenges included difficulty with using delirium screening tools, an organizational culture not conducive to delirium prevention, and competing clinical priorities. Proposed solutions were also discussed, including decision-support systems with automated pager alerts and associated delirium order sets, which may help improve delirium care coordination and standardization.

**Conclusion:**

At a major university hospital, nurses affirm the difficulty experienced with delirium screening and identification, particularly due to screening tool challenges, cultural barriers, and clinical workload. These impediments may serve as targets for a future implementation trial to improve delirium screening and management.

**Supplementary Information:**

The online version contains supplementary material available at 10.1186/s12913-023-09681-4.

## Introduction

Delirium is a major complication of hospital admission that is associated with increased length of hospitalization [1], cognitive and functional decline [2, 3], and substantial healthcare costs [4]. Older patients are particularly vulnerable to delirium, which may ominously predict future Alzheimer’s Disease and Related Dementias [5]. Preventing, identifying, and managing delirium are thus important elements of optimizing clinical care for older, vulnerable patients during hospitalization.

Unfortunately, delirium screening tools used in routine clinical practice demonstrate low sensitivity (~ 30%) with delirium detection [6], despite validation studies indicating sensitivities > 80% [7]. At our own institution, we found that the documented delirium detection rate was < 5% on major medical and surgical inpatient units during an electronic health record audit. Conversely, trials and observational studies conducted on these units reveal a delirium incidence of approximately 20% during inpatient admission based on trained research team assessment [8–10]. This incongruence may be explained by challenges related to implementation of delirium screening tools. In fact, nurses screening for delirium in the emergency department and intensive care units often report major barriers, including lack of training with screening tools, demanding workload, and competing clinical demands [11, 12]. It is unclear, however, whether these same challenges apply to nurses on inpatient non-intensive care units, or if there are additional, distinct barriers present in this setting. As delirium is associated with adverse clinical events during hospitalization (e.g., falls, prolonged admission) and increased healthcare expenditures [1, 4, 13, 14], there is a critical need to improve delirium detection during inpatient admission, such that evidence-based interventions can be implemented to mitigate risk of adverse consequences.

The objective of this study was to identify barriers and challenges to delirium screening on inpatient units (non-intensive care) at a major university hospital. The rationale for focusing on these units is that delirium often occurs in this setting, and prior implementation studies have largely centered on intensive care units and the emergency department [11, 12, 15]. The approach in the current study was to perform a qualitative analysis of nursing focus groups from two representative medical and surgical units that typically perform delirium screening and identification. The results will ultimately inform the design of a future quality improvement initiative for optimizing delirium screening and management strategies on inpatient units.

## Methods

### Study Design and Overview

This was a pre-implementation diagnostic evaluation study [16], which sought to identify challenges, barriers, and facilitators to delirium screening and management at a major university hospital. The study followed a qualitative design, as focus groups were conducted to identify challenges associated with delirium screening and management from nursing perspectives, as daily delirium screening is performed by nurses at our institution. Focus groups were chosen as the qualitative technique for multiple reasons. First, the group setting enables rich, dynamic conversation and exchange of ideas that centers around a shared process or experience (i.e., delirium screening and management). Second, the group format allowed for a broad, diverse array of opinions and experiences, which is important for understanding all aspects of delirium care. Last, scheduling of focus group meetings (during other, routinely scheduled nursing meetings) was logistically easier than attempting to schedule several independent interviews given clinical scheduling constraints. All operations were conducted at Michigan Medicine (Ann Arbor, MI USA), and institutional review board exemption was obtained the University of Michigan Medical School (HUM00205546). As such, the requirement for written consent was also waived. This study was also conducted in accordance with the Standards for Reporting Qualitative Research (SRQR; Supplemental Content 1) [17].

### Eligibility and recruitment

All nurses working within two major medical-surgical units at Michigan Medicine were eligible to participate in the focus groups. These are two of the primary non-intensive care units at our institution (University Hospital, Michigan Medicine) that care for non-cardiac surgical patients and medical inpatients. Nurses routinely screen for delirium on these units using the Confusion Assessment Method (CAM), though the charted delirium incidence is low (< 5%), and nursing leadership on these units welcomed the focus group initiative to better understand challenges nurses face with delirium screening and management. Utilizing a convenience sampling approach, we invited all nurses working on these two inpatient units to participate. As we sought to elicit a diverse range of perspectives, any nurse providing care in one of the two units were eligible regardless length of time working on the unit or years in practice as a registered nurse.

Focus groups were conducted via email invitations and nurse manager communications during in-person and virtual meetings. Final recruitment reflected a convenience sample of available nurses who agreed to participate; there was no mandate to participate. Informed consent was waived based on institutional review board exemption previously mentioned.

### Data Collection

#### Focus Groups

Using the Consolidated Framework for Implementation Research [18, 19], which is commonly used for systematically assessing potential barriers and facilitators to implementation of an intervention, the team created a focus group interview guide that addressed four areas: (1) general perceptions regarding delirium; (2) barriers to delirium screening; (3) challenges with delirium management; and (4) factors that would facilitate delirium screening and management (Supplemental Content 2). Field notes were taken after each session.

### Procedures

Focus groups were held with available unit nurses, aiming for 5–10 participants per group. Focus group participants represented a nested sample from all unit nurses eligible for participation (n = 127). Participants expressing interest in participation received a primer email prior to the focus group session, which summarized the objective of the study and focus group. To facilitate participation, focus groups were held in a hybrid format – a video conference link was provided, and in-person attendees participated from a private room. Focus groups were conducted – and coded – by study team physicians (one male, one female) with prior focus group experience (J.R. and P.E.V.) using previously described methods [20]. These physicians lead a clinical research program that aims to optimize neurocognitive health in hospital inpatient settings. Participants did not have a working clinical relationship with the two group leaders (J.R. and P.E.V.). Focus groups were audio recorded and transcribed verbatim. No one was present for these sessions other than group leaders and focus group participants. While transcripts were not provided, general feedback will be presented during future nursing meetings for subsequent implementation research protocol development. Focus groups were held until signs of thematic saturation were present (n = 3 sessions).

### Statistical analysis

The sample size for this study was based on convenience samples from Michigan Medicine medical and surgical units. There was no predetermined primary outcome for this study, as the objective was to identify barriers and challenges with delirium management from nursing perspectives. As such, no power calculations were required for this study design.

**Fig. 1:**
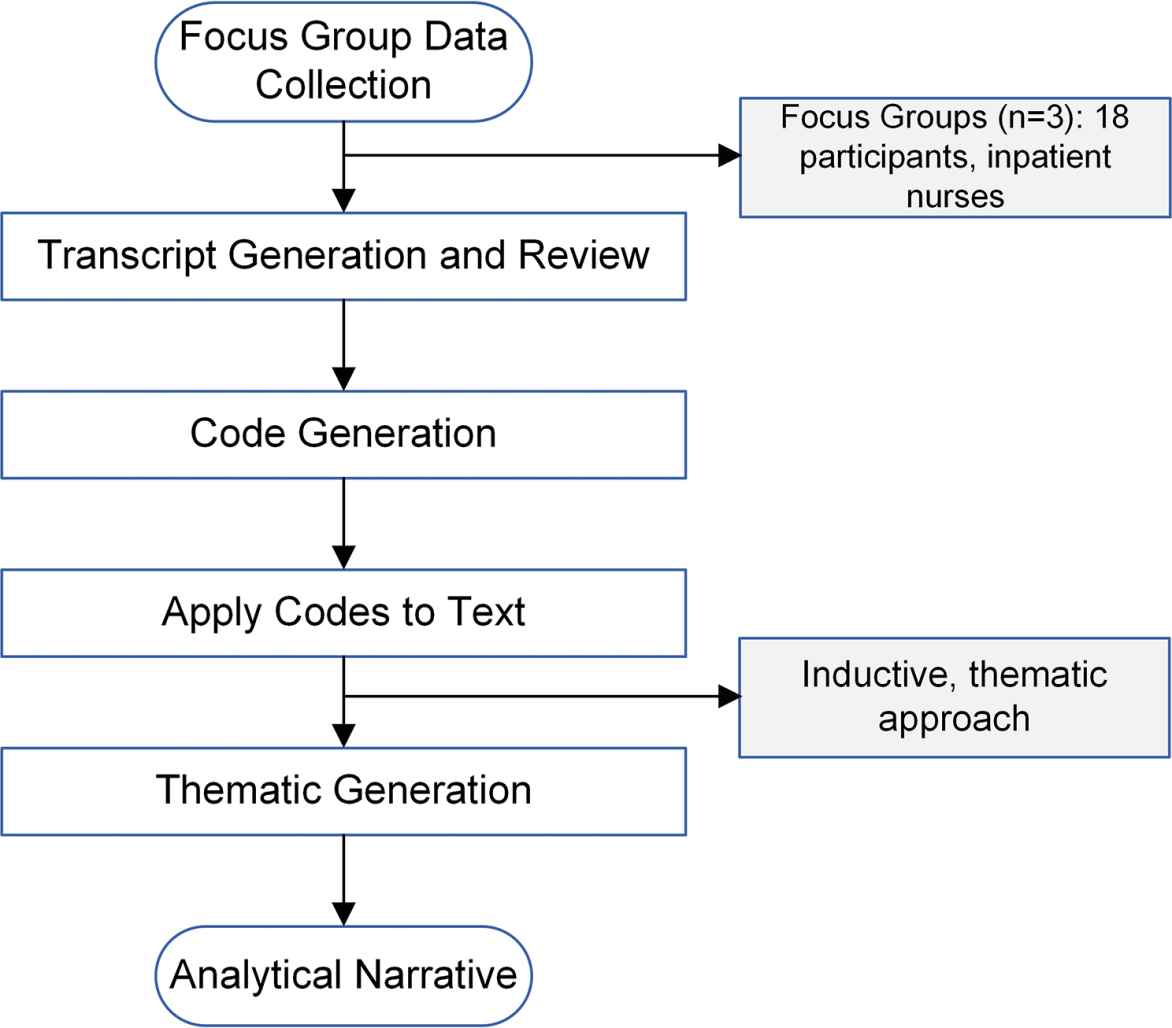
Qualitative Study Design Study flow diagram presented. Three focus groups were conducted with 18 inpatient nurses from the medical-surgical units chosen for study participation. After focus group completion, transcripts were generated and reviewed. Codes were generated and incorporated into a main codebook, after which codes were applied to the text to generate major study themes. Themes were then incorporated into an analytical narrative, for which findings were compared to similar, related studies (see Discussion section)

Focus group data were analyzed via inductive thematic analysis [21]. That is, no predetermined theories or structures were used to analyze the data – the results guided the analytical structure. In brief, this technique involves a six-phase process for completing the analysis: [21] (1) familiarization with the data via multiple, detailed reviews of transcripts; (2) generating brief labels (“codes”) to identify pertinent transcript data that relates to the original research question; (3) initial theme generation based on the codes developed; (4) reviewing of initial themes multiple times against the dataset; (5) defining and naming of final themes after final revisions; and (6) writing the analytical narrative by weaving together the extracted themes and contextualizing the analysis in relation to prior, related investigations (Fig. 1). The two coders (J.R. and P.E.V.) reviewed transcripts together with a consensus approach for coding, and MAXQDA software (VERBI Software, 2021) [22] was used to facilitate data coding, analysis, and thematic generation.

## Results

### Qualitative Focus Group results

Three focus groups were conducted with 18 nurses with a median (interquartile range) of 3.5 (2.8–5) years of experience on their current inpatient unit. Thematic saturation was present by the third meeting. Focus groups lasted a mean (± standard deviation) duration of 42 (± 8) minutes. Participants shared their experiences, insights, and suggestions with identifying and managing delirium in the hospital inpatient setting.

Delirium is commonly encountered by nurses on inpatient units, particularly in the early postoperative period and in the context of sleep deprivation. Nurses report negative experiences, often feeling frustrated by the challenges surrounding delirium care, as illustrated by major themes identified (Table 1).

**Table 1:** Major Themes Surrounding Inpatient Delirium Care – Nursing Perspectives

Theme	Major Barriers or Action Items Identified
1. Delirium Screening Challenges and Perceptions	● Determining acute change from baseline
	● Screening tool education and training
	● Subjectivity with delirium assessment
	● Inaction with positive screens
2. Organizational Culture Towards Delirium	● Dismissive attitudes
	● Lack of delirium management knowledge
	● Delirium as a priority with hospital leadership
	● Hospital environment – sleep interruptions
	● Lack of standardized approach to delirium
3. Competing Clinical Priorities	● Contemporaneous clinical demands
	● Charting fatigue
4. Desired Improvements	● Decision support systems (e.g., pager alerts)
	● Delirium prevention and management order sets
	● Multidisciplinary collaboration
	● Standardized, recurrent delirium education

### Theme 1: Delirium Screening Challenges and perceptions

Nurses reported many challenges with using delirium screening tools. Identifying acute cognitive changes from baseline is a core component of the screening tool used (CAM) [23], but nurses expressed considerable difficulty determining if a patient’s cognitive status represents an acute change or baseline impairment. This information is not easily accessible to nurses due to lack of family caretaker presence at baseline and unreliable documentation of cognitive status. As such, without knowing baseline cognition, nurses do not feel comfortable completing delirium screening forms.

*“Well, the problem with screening is, if we don’t have a true baseline for the patient, that can make screening hard…”* (Participant 18).

There is also confusion regarding the instructions for properly completing CAM screening. Participants did not consistently receive prior CAM training, and the need for additional education was discussed.

*“When it came to our CAM tool…I don’t necessarily know that someone sat down with me and went through each step of it, but it was just more of an expectation that I knew I need to chart [it] every night”* (Participant 7).

Additionally, elements of the CAM, such as inattention, are often scored subjectively, in a non-standardized manner. The perception of inattention – and other elements of delirium – may also depend on the level of nursing experience and education. This leads to variable interpretation and scoring.

“*I think that a lot of the CAM scoring is dependent on the person who is doing the scoring. It can be very…individualized based on the perception of the individual”* (Participant 3).

Lastly, delirium screening is perceived to be a low-value activity by some nurses. This is because positive delirium screens do not trigger any alerts or interventions; in fact, positive screens generally do not lead to a change in clinical management.

“*Once someone screens positive for delirium, nothing happens after that. [With sepsis], the charge nurse gets a page, the nurse gets a page, [the] doctor…that’s with the sepsis screening. There’s nothing like that that exists with the delirium.”* (Participant 5).

*“If you don’t show me the added value, I’m not doing it. I’ve got enough stuff that I’ve got to do, that I’m not going to focus on this because I don’t see the value added in it. So, to get buy-in from your nurses to do anything, you have to show the value. And so, you may tell me you’ve got to document this, but if I know it’s not going to make a difference in the care that’s being provided to my patient, I don’t see the added value”* (Participant 11).

### Theme 2: Organizational Culture towards Delirium

Nurses described indifferent attitudes towards delirium by different healthcare professionals and health system leaders. Physician interactions were described, at times, as counterproductive. Specifically, nurses have experienced pushback and/or dismissive attitudes when reporting information relating to delirium.

*“As a nurse, we’re taught to report changes with your patients…But if you get shot down by a doctor – ‘that wasn’t important’ – or whatever the case may be, then a lot of the times nurses don’t want to bring anything else up…”* (Participant 15).

Alternatively, nurses may encounter engaged, supportive physicians; however, these tend to be junior residents who may not have the knowledge, experience, or training to adequately management delirium.

*“In reality, we’re working with interns sometimes that are like, ‘well…I don’t know what the appropriate delirium orders are. I don’t know what we should be doing with that’”* (Participant 1).

From the standpoint of hospital leadership, delirium is not perceived to be a high-level priority, nor is it a quality measure throughout the hospital. By extension, there is a lack of accountability for delirium screening and management.

*“I feel like there’s less of an investment from… executive leadership…because it’s not directly tied to a quality measure. You know, it is not a [hospital-acquired infection], but really it is affecting length of stay. So, I think as much as we can get buy-in from executive leadership…”* (Participant 3).

The hospital environment itself is not conducive to delirium prevention. Nurses described constant nightly interruptions for patients: evening and early morning blood draws, checking vitals, and other clinical services and tasks that keep patients up at night. Sleep deprivation is thus perceived to be a contributing factor to delirium risk.

“*I do think we are a major contributor. The turning, the vitals signs, medications [at] all times…maybe we all need to do a better job… Then we tell the patient, ‘well, you’re not at the hospital to sleep.’ I would be delirious too.”* (Participant 14).

Finally, nurses described the lack of a standardized approach to delirium management. Different hospital services have different approaches to delirium, and there is a lack of care coordination among services caring for a patient. Nurses perceive that improved coordination could lead to an integrated, aligned plan for mitigating delirium risk. Additionally, increased pharmacy involvement in day-to-day patient care is desired as a central component to such multidisciplinary care integration, as this may reduce deliriogenic medication administration.

“…*When we get psych involved and geriatrics involved, they are like two vastly different teams, and they both have vastly different approaches*” (Participant 2).

*“I definitely think it would be beneficial, if we have people that are screening positive, [to] do an interdisciplinary team meeting. So, everybody meets together, pharmacy meets…I don’t understand why we don’t do this anyway.*” (Participant 7).

### Theme 3: competing clinical priorities

Nurses commonly report a substantial clinical workload during shifts, which can serve as a barrier to successful delirium management. Interventions recommended for delirium management are also labor-intensive and often unrealistic given the concurrent clinical demands when caring for multiple patients.

*“The interventions they [physicians] suggest – while they’re rooted in best practice and research – [they] can take a full 1:1 nurse intervention…that is not always realistic when you have three or four other high-acuity patients”* (Participant 3).

In this context, charting fatigue is a major problem given the workload burden nurses experience. Retroactively charting delirium encounters becomes particularly challenging.

*“Who wants to go back and do all that charting? I haven’t even finished charting on my other 3 patients...”* (Participant 15).

As a consequence of competing clinical priorities, nurses sometimes default to interventions such as restraints and pharmacological sedation to lessen the workload associated with delirious patients. Nurses may feel comfortable and emboldened by this approach knowing that injury is less likely.

*“When they’re restrained, you know they’re not going to be on the floor at least. So, unfortunately, those easier interventions like polypharmacy…(are) less time consuming, less taxing, like restraints*…” (Participant 3).

### Theme 4: desired improvements

Decision-support strategies, such as real-time alerts and pages, could be helpful for raising delirium awareness among clinicians and services. These alerts could also serve as a prompt to trigger delirium care pathways with positive screens.

*“What if [an alert] could just serve as an initial reminder, like, ‘you’re CAM positive, here’s your reminder - check with your physician about initiating delirium protocols?’”* (Participant 16).

*“Yeah, if [a positive delirium screen] triggered, ‘initiate delirium bundle,’ and we sort of knew what that meant and what to do about it, that would be really helpful” (Participant 6).*

Intervention order set bundles, specifically for delirium prevention and management, were proposed by nursing participants. These bundles could be triggered by discrete events, such as inpatient admission (prevention bundle) or positive CAM screens (management bundle). Bundles could also integrate pre-existing hospital resources.

*“So, you know, if [there was] … a pathway, right? Or a bundle? ... Are we re-orienting, reducing medications when possible; are we, you know, doing everything that we can? Putting them on sleep protocol, changing lab times, you know. Those are things that are really simple that might have a big impact on our patients that are at-risk”* (Participant 3).

Multidisciplinary meetings, including family members or caretakers, pharmacy, social work, and other supporting services are desired. Family involvement would be particularly helpful for understanding baseline patient function.

*“Meet with everyone else that’s consulted. Meet with social work, get family involved so we actually know what baseline is...” (Participant 7).*

Lastly, nurses endorsed the desire for recurrent education on various delirium topics.

In particular, education on different delirium phenotypes (e.g., hyperactive, hypoactive) would be welcomed, as nurses report that hypoactive delirium is often likely missed or unrecognized.

*“We could really use a tool to educate us on hypo- versus hyper-[active delirium]…”* (Participant 15).

*“I feel like if you’ve never been educated on it, though, it’s very easy to miss, right? And like, we’ve had a lot of new staff, I feel like we’re probably missing that piece… And I think that looking at both aspects – the hyperactive versus hypo – right? A lot of times we see more the hyper than the hypo, and it’s so hard to decipher…”* (Participant 14).

Lastly, additional education would be welcomed with CAM screening, as nurses do not consistently receive background training in the CAM. In fact, delirium educational sessions are unit-based and non-standardized throughout the hospital.

*“I know that myself – and I’m sure other people – don’t feel really confident in both the assessments…I think having some formal education on it, and the steps that we take once it’s identified, would make it less maybe daunting to officially [identify delirium] and start the process of helping this patient resolve their delirium”* (Participant 6).

## Discussion

Hospital delirium is a major public health concern, particularly given the associated morbidity, mortality, and aging populations. Through focus group and survey analysis, we identified distinct challenges that nurses commonly experience with delirium assessment and management in the inpatient setting. Notably, nurses endorsed multiple screening tool challenges, including how to assess and report acute changes from baseline, non-standardized methods for assessing cognitive domains, and the need for additional training with delirium screening tools. The value of delirium screening was also called into question, as positive delirium screens do not elicit subsequent action at our institution. Daily clinical workload also serves as a barrier to optimizing delirium screening and management, and nurses do not perceive adequate support from hospital leadership for performing delirium screens. Nurses voiced support for decision-support systems, such as automated pagers and alerts, that could link to delirium order sets within the electronic health record. These strategies may help to raise delirium awareness and standardize delirium care throughout the hospital.

These findings are consistent with prior studies and quality initiatives that have sought to identify challenges with delirium recognition and management. A previous quality improvement initiative at a large teaching hospital revealed that delirium was frequently unrecognized by clinicians prior to hospital discharge [24]. Based on survey reporting, lack of delirium education, the fluctuating nature of delirium, and presence of the hypoactive subtype likely contributed to confusion and poor recognition. Likewise, similar quality and implementation studies in the emergency department and intensive care unit settings have revealed that nurses commonly report feeling overwhelmed, uncomfortable with delirium screening tools, and perceiving that delirium identification was not a high cultural priority [11, 12]. Nurses identified with these same themes at our institution in the inpatient setting. Furthermore, barriers involving delirium screening, systems-based hospital dynamics, and proposed management strategies (e.g., decision-support systems, delirium order sets) could serve as targets for a future implementation program to improve delirium care for hospital inpatients.

This study also builds upon previous findings to provide additional insight into the challenges that nurses face. Both in this study and prior investigations [11, 12, 25], nurses have reported lack of comfort and/or inadequate training with delirium screening tools. Based on focus groups from this study, nurses voiced considerable difficulty with identifying acute changes in cognition from baseline. This challenge stems from episodic interactions with patients, unknown pre-admission cognitive status, and, particularly in the COVID era, inconsistent family member (or care partner) availability at the bedside [26]. Algorithms that assess for acute changes during the course of hospital admission may thus be helpful to nurses while trying to assess for fluctuations in cognition [27]. Automated, simplified algorithms may also mitigate confusion that exists with screening tool instructions by providing an objective, standardized way of assessing key cognitive domains (e.g., attention) [28]. Recurrent, standardized educational sessions may also increase confidence and improve performance with delirium screening tools. While contemporaneous clinical demands may limit delirium screening, targeted decision-support systems, such as pager alerts and pre-populated order sets, may ease workload by calling attention to high-risk patients, facilitating clinical decision-making, and referring patients to delirium prevention programs (e.g., AGS CoCare: HELP program) [29]. Nurses also voiced a perceived lack of care coordination among consultant and ancillary services (e.g., pharmacy), and delirium is also managed on a unit-by-unit basis without standardized hospital protocols. Optimized nurse staffing ratios may also help to implement appropriate, evidence-based delirium interventions, particularly for high acuity patients. Finally, nurses also posit that delirium may garner more attention with hospital leadership if it formally served as a quality measure. These considerations can serve as targets for future quality improvement and implementation studies.

There are multiple study limitations to consider. First, the data were derived from a convenience sample of nurses, based on availability. Nurses willing to participate in survey and focus group sessions may provide different responses compared to those who did not engage in such quality improvement efforts. As such, sampling bias may have been present, as results generated may not accurately reflect the breadth of perceptions and experiences from nurses on the medical and surgical units sampled. Lastly, data were also derived from a single hospital system, as other hospital systems that were solicited for participation were unavailable.

In summary, this study highlights major challenges encountered by inpatient nurses with respect to delirium recognition and management. These findings highlight the need for standardized, evidence-based delirium care pathways, and a future implementation trial could target the barriers identified for optimizing clinical practice related to delirium.

## Electronic supplementary material

Below is the link to the electronic supplementary material.

Supplementary Material 1

Supplementary Material 2

## Data Availability

The datasets used and/or analyzed during the current study are available from the corresponding author on reasonable request.
