# Supplementary material for: Barriers to delirium screening and management during hospital admission: a qualitative analysis of inpatient nursing perspectives
Source: BMC Health Serv Res. 2023 Jun 29;23:712. doi: 10.1186/s12913-023-09681-4 (PMC10311863; doi:10.1186/s12913-023-09681-4)
Supplement: Supplementary file 2 — Supplementary Material 2 [file 12913_2023_9681_MOESM2_ESM.docx]

**Delirium Focus Group Discussion Guide – Nursing Participants**

**Introduction statement:** To begin, I’d like to thank you for agreeing to participate in this focus group today. To help us with the discussion, I have included Drs. …, …. They have run focus group sessions like this before and may ask a few questions as well.

As you may have heard, we are interested in finding ways to reduce delirium in hospitalized patients. We’ve been thinking a lot about possible interventions to pilot, but we realized we first needed to understand (1) how delirium is identified on the hospital units and (2) challenges encountered with delirium screening (and delirium care more broadly). You all have such valuable experience with delirium screening and assessment, so we’re really excited to hear your thoughts.

**Process Overview:** So, during the focus group today, we will be talking about your thoughts and experiences with trying to screen for delirium in your patients. In particular, we want to understand:

(1) Your general thoughts about delirium

(2) Strategies used on your floor for delirium screening and detection

(3) Ultimately, shortcomings with delirium screening and delirium care more broadly

The main objective is to gather information – **we are not trying to reach a group consensus**. We just want to learn, and your experiences, views, and opinions are extremely valuable. I just want to stress that your participation is **completely voluntary**, and the discussion is **strictly confidential**. The information here is for qualitive improvement purposes, and nothing will be shared outside of this group. Furthermore, there will be judgement. We hope to generate a rich discussion and generate information – nothing will be shared, no judgement.

We do record this session, but this is purely for qualitative improvement purposes and to make sure we do not miss anything. Everything will be de-identified, and the transcript will be destroyed after the study is complete.

Ultimately, the goals are to design a system to improve delirium screening in the hospital and (2) ultimately, improve delirium management in the hospital more broadly.

**Ground Rules**

- The discussion will last approximately one hour
- Feel free to get up as needed (e.g., restroom break, refreshments)
- Speak as freely and opening as you feel comfortable. We want to hear from everyone.
- No detail is “too small” or insignificant to share—we want to learn as much as we can
- **No judgement or repercussions** – all discussion will remain anonymous
- Everything that is said in here should stay in here.
- Everyone should participate
- Turn off cell phones if possible

Final questions before we start?

****Recording Begins****

|  |  |
| --- | --- |
| *Opening* | To get started, I thought we could go around and share how long each of you has been a practicing nurse? What types of patients do you care for on the wards? |
| *Introduction* |  |
| *Transition* | Is delirium something that you think about when taking care of patients? Any particular contexts?  Understanding of delirium? |
| *Key Questions (4-6)* | - What kind of education have you received with delirium? |
|  | - What strategies do you currently use for delirium screening?   (*set time, prompts?*)   - What problems, if any, do you encounter with these screening practices (*e.g., conduct, time, interpretation, scoring, charting)*? - How often does screening take place? - Do you receive education/training with these screening procedures? |
|  | ● What could be done to improve delirium screening? |
|  | ● What happens once a patient screens positive?   - Any protocols/care pathways that are activated? |
| *Ending* | So, to wrap up, what are the most important aspects of delirium on the units that need attention? |
|  | Anything I’m missing that we didn’t discuss? |

**Helpful phrases to get more information**

*Can you tell me more about that?*

*Could you tell me why?*

*Could you tell me what that looks like?*

*Could you give me an example?*
